# Genetic diversity and signatures of selection in BoHuai goat revealed by whole-genome sequencing

**DOI:** 10.1186/s12864-023-09204-9

**Published:** 2023-03-15

**Authors:** Zhi Yao, Shunjin Zhang, Xianwei Wang, Yingwei Guo, Xiaoling Xin, Zijing Zhang, Zejun Xu, Eryao Wang, Yu Jiang, Yongzhen Huang

**Affiliations:** 1 Key Laboratory of Animal Genetics, Breeding and Reproduction of Shaanxi Province, College of Animal Science and Technology, Northwest A&F University, No. 22 Xinong Road, Yangling, 712100 Shaanxi China; 2 Henan Provincial Animal Husbandry General Station, Zhengzhou, 450008 Henan China; 3 Institute of Animal Husbandry and Veterinary Science, Henan Academy of Agricultural Sciences, Zhengzhou, 450002 Henan China

**Keywords:** BoHuai goat, WGS, Genetic diversity, Population structure, Selection

## Abstract

**Background:**

Cross breeding is an important way to improve livestock performance. As an important livestock and poultry resource in Henan Province of China, Bohuai goat was formed by crossing Boer goat and Huai goat. After more than 20 years of breeding, BoHuai goats showed many advantages, such as fast growth, good reproductive performance, and high meat yield. In order to better develop and protect Bohuai goats, we sequenced the whole genomes of 30 BoHuai goats and 5 Huai goats to analyze the genetic diversity, population structure and genomic regions under selection of BoHuai goat. Furthermore, we used 126 published genomes of world-wide goat to characterize the genomic variation of BoHuai goat.

**Results:**

The results showed that the nucleotide diversity of BoHuai goats was lower and the degree of linkage imbalance was higher than that of other breeds. The analysis of population structure showed that BoHuai goats have obvious differences from other goat breeds. In addition, the BoHuai goat is more closely related to the Boer goat than the Huai goat and is highly similar to the Boer goat. Group by selection signal in the BoHuai goat study, we found that one region on chromosome 7 shows a very strong selection signal, which suggests that it could well be the segment region under the intense artificial selection results. Through selective sweeps, we detected some genes related to important traits such as lipid metabolism (*LDLR*, *STAR*, *ANGPTL8*), fertility (*STAR*), and disease resistance (*CD274*, *DHPS*, *PDCD1LG2*).

**Conclusion:**

In this paper, we elucidated the genomic variation, ancestry composition, and selective signals related to important economic traits in BoHuai goats. Our studies on the genome of BoHuai goats will not only help to understand the characteristics of the crossbred but also provide a basis for the improvement of cross-breeding programs.

**Supplementary Information:**

The online version contains supplementary material available at 10.1186/s12864-023-09204-9.

## Introduction

Goats were one of the first domesticated domestic animals and the most adaptable and geographically widespread of domestic animals [1]. Compared with cattle, only 10% of mtDNA variation in goat breeds is spread across continents (Europe/ Africa/Asia/Middle and Near East), compared with more than 50% in cattle [2]. This weak structure suggests that goats experienced extensive intercontinental gene flow, suggesting their importance in human history for migration and commerce. At present, most scholars believe that the direct ancestral group of the present domestic goat is the bezoar (*Capra aegagrus)*, and it spread after domestication in the Middle East centers [3–5]. According to the genome-wide analysis of domestic goats, domestic goats worldwide can be divided into four continental groups: EUR (European) 、AFR (African) 、SWA-SAS (Southwest Asian), and EAS (East Asian) [6]. Based on whole-genome sequencing, many studies have focused on economic traits related to goat genetics and production performance, such as litter size traits, heat resistance, and disease resistance [7, 8]. These findings demonstrate the potential of genomes in goats that are important for agricultural development, which in turn can be used in the selection of goat breeds for adaptation to the environment and domestication [9]. The mining of these good genes could lead to better genetic breeding strategies to improve the adaptability and productivity of goats [10, 11].

The most comprehensive collection of individual genetic variation is provided by whole-genome sequencing, which can be used to study population structure and identify polymorphisms that could influence livestock’s economic attributes [12]. WGS provides a better insight into genetic diversity and genomic footprints under positive selection [13]. The boer goat is a famous mutton breed, its fecundity, disease resistance and genetic stability are higher than other breeds [14]. Therefore, Boer goat has been introduced into many countries to improve local goat breeds [15]. In Shenqiu County, Henan, China, a new breed named Bohuai goat was developed by crossing the introduced Boer goat with the local Huai goat. Cross-cross fixation was performed after undergoing three generations of progressive hybridization. After more than 20 years of cross improvement, it’s superior to the male parent and the female parent in terms of meat production rate, reproduction rate and meat quality. BoHuai goat’s performance in all aspects has been greatly improved. Most previous studies have focused on the meat performance of the BoHuai goat [16, 17]. There has been very little research on the genome of the BoHuai goat. In this study, we used whole genome sequencing to to identify important genes in BoHuai goats, as well as an in-depth understanding of the genetic structure of BoHuai goats is crucial for improving future breeding of Bohuai goats.

To achieve this goal, we sequenced the full genomes of 5 Huai goats and 30 BoHuai goats, combining data from 126 published goats from around the world to detect genetic variation, population structure, and selective scanning. Our results will lay a foundation for further studies on the genetic basis of important economic traits and provide ideas and basis for future improvement in BoHuai goats.

## Results

### Sequencing and variants detection

A total of 4,810,042,888 reads were generated after genome sequencing from 30 BoHuai goats, with an average depth of 8.16X (Table S1). To place BoHuai goat into a global context, we also analyzed the genomes of other goat populations worldwide. The other populations (Table S2) include European goat, African goat, Middle East goat, different goat breeds in north and south China, and Boer goat and Huai goat. All of the clean reads were aligned to the *Capra hircus* reference genome (ARS1) using Sentieon [18] software. SNP analysis was performed using GATK. We identified 14,847,751 biallelic SNPs in 30 BoHuai goat. Of these SNPs, functional annotation of polymorphic loci showed that the majority of SNPs existed in the intergenic region (59.6%) or exonic region (37.4%). Exons accounted for 0.77% of the total SNPs, including 71,193 non-synonymous SNPs and 99,811 synonymous SNPs (Table S3).

The total number of single nucleotide polymorphisms detected varied from 14 to 18 million for different breeds (Table S3). Middle East goat (18,237,170) has the highest number of SNPs, followed by Tibetan goat (17,767,153), Chinese northern goat (17,534,707), Africa goat (16,851,639), Chinese southern goat (16,287,038) and European goat (16,283,645). However, the number of SNP in Huai goats are lowest. The number of SNP in BoHuai goat was between Boer goat and Huai goat.

### Population structure analysis

**Fig. 1:**
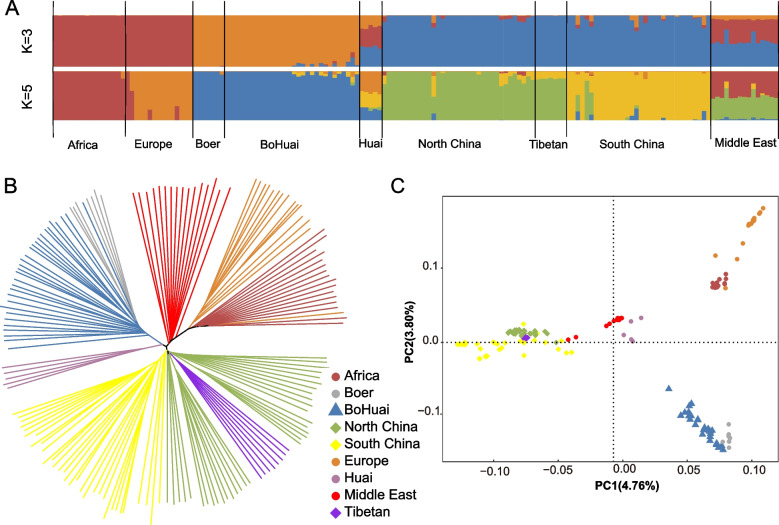
Population structure of BoHuai goats and its relationship with other breeds in the world. **A** ADMIXTURE was used with K = 3 and K = 5 for model-based clustering among different goats. Colour and label them by geographical area. Neighbor-joining trees (**B**) and principal component analysis (**C**) separated the goat breeds (161 animals in total) into nine categories

To explore the genetic relationships between the BoHuai goat and other goat breeds around the world, genomic SNPs were used for ADMIXTURE, neighbor linkage (NJ), and principal component analysis (PCA) (Fig. 1). The first and second PCs explained 4.76 and 3.80% of the variation in the entire genetic data, respectively. The analysis revealed clear geographical patterns of goat distribution. The goats from Africa and Europe clustered together, and the goats from the north and south of China and Tibet clustered together. And the Boer and the BoHuai goats stay together, but the Huai goats are in groups of their own. The ancestry proportions of individuals in different goats inferred by the ADMIXTURE are presented in Fig. 1 A. As can be seen from the figure, these goats can be divided into different ancestors at K = 3 or K = 5, but Boer goats and Boer goats have always had very similar ancestral components. BoHuai goat composition is more similar to Boer goat which indicates that the genetic influence of the Boer goat was greater on the BoHuai goat than that of the Huai goat. The phylogenetic tree (Fig. 1B) and PCA (Fig. 1C) showed similar results. All different groups form independent clusters. The individuals of the BoHuai goat are also mostly clustered together and close to the Boer goat.

### Genomic variation, genetic diversity, and linkage disequilibrium

**Fig. 2:**
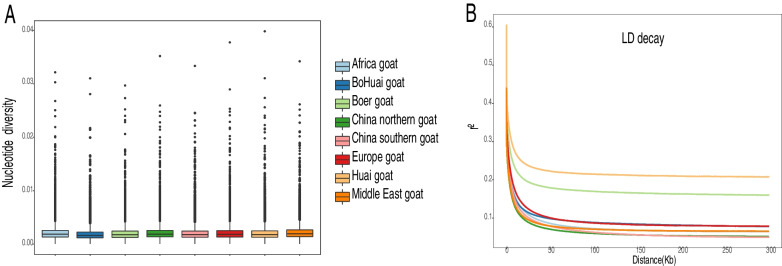
**A** The nucleotide diversity of goat breeds from different regions. The black line in the boxplot is the median line and the outside points are outliers. **B** Genome-wide average LD decay is estimated from each categorie. Different colored lines represent different category. The legend in the middle is shared by both figures

To look at the variation in these different goat populations (EUR、AFR、SWA-SAS、EAS), we calculated their nucleotide diversity. The results showed that the nucleotide diversity of different populations was similar, with a median of about 0.002. Notably, the nucleotide diversity of Middle Eastern and north Chinese goats was higher than that of other breeds (Fig. 2A). From the perspective of linkage disequilibrium (LD) (Fig. 2B), there are some differences among various groups. At distances between markers (> 50 kb), the Middle East and northe China had the lowest LD levels, with the highest LD levels being Locust goats, followed by Boer goats and BoHuai goat.

### Comparison of SNP in BoHuai goat, Huai goat, and Boer goat

We compared the common and the nonsynonymous SNPs (nsSNP) loci in Boer, Huai, and BoHuai goats. (Fig. S1). The SNP shared by the three breeds were 9,903,772. The common SNP of BoHuai goat and Boer goats were 12,829,527, accounting for 86.4% of BoHuai goat SNP and 79.1% of Boer goat SNP respectively. The common SNP of BoHuai and Huai goat was 10,844,692, accounting for 73.0% of BoHuai goats and 74.1% of Huai goats, respectively. The results indicated that the abundant SNP genetic resources in the BoHuai goat mainly came from the lineage of the Boer goat. By comparing the nsSNPs of Boer and Huai goats, we obtained 11,552 and 10,975 specific nsSNPs in Boer and Huai goats, respectively. Following the strategy of other studies, we looked for genes with more than five nsSNPs in each breed. Finally, a total of 280 and 236 genes were identified in Boer and Huai goats. Among these genes, using DAVID gene ontology, 202 and 235 significant (*P* < 0.05) GO BP terms were enriched in Boer goat and Huai goat, respectively. (Figs. S2, S3 and Tables S5, S6). The GO enrichment analysis revealed that most genes are related to amino acid transport and cell homeostasis.

### Signatures of selection

**Fig. 3:**
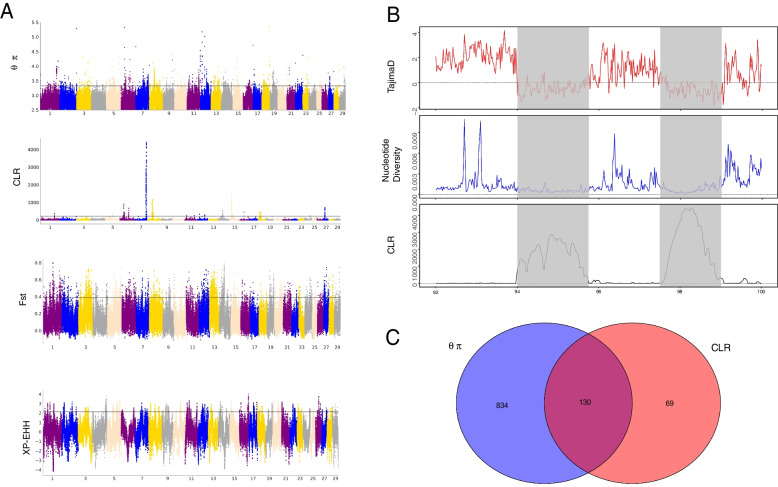
Analysis of selection characteristics of BoHuai goats. **A** The Manhattan diagram shows the situation of a selective sweep in BoHuai goats. **B** Nucleotide diversity, CLR, and Tajima’s D plot of the 92-100 M genomic region of chromosome 7. **C** Number of candidate genes supported by θπ and CLR methods in each of the Venn diagram components in BoHuai goats

Nucleotide diversity analysis (θπ) and complex likelihood ratio (CLR) were used to detect genomic regions associated with selection in the BoHuai goat population**.** And we selected the top 1% of signals as candidate regions. 964 genes were screened by θπ (Table S7), and 199 genes were screened by CLR (Table S8), as shown in Fig. 3A. A total of 130 candidate genes were obtained by the intersection of the two methods (Fig. 3C). These genes were mainly distributed on chromosomes 7, 8 and, 27, and a large number of candidate genes were clustered in the 94 M–99 M region of chromosome 7. We used the KEGG pathway and gene ontology (GO) to perform a functional enrichment analysis of these overlapping genes. The results showed that the KEGG pathway was significantly enriched as “Cholesterol metabolism”, which contained three genes (*LDLR*, *STAR*, *ANGPTL8*)(Table S9). Results of GO terms show that these genes are significantly enriched in “negative regulation of neuron differentiation”, “cytoplasm”, “Positive regulation of mitotic cell cycle”, and “Phospholipid Metabolist Process”, etc. (Table S10).

We also used F_ST_ and XP-EHH methods to compare selection between Boer goat and BoHuai goat populations. By F_ST_ and XP-HH methods, 1134 and 393 genes were detected, respectively (Tables S11 and S12). The intersection of the two methods was also used to obtain 138 candidate genes. We also used the KEGG pathway and GO (Gene Ontology) for functional enrichment analysis of these overlapping genes. The results showed that the most significant enrichment pathway was the “Focal adhesion” (Table S13), including six genes: *ITGB4*, *COL4A4*, *CCND2*, *GRB2*, *COL4A3*, and *FLNB*, which are associated with reproductive traits.

## Discussion

Genetic diversity is important for the understanding of environmental adaptability of livestock and poultry and the intuition of conservation and utilization of breed resources. In this study, we found that different breeds of goats in different regions maintained similar levels of genetic variation. This situation is consistent with previous reports and may be due to goats not undergoing the same high selection as cattle do [19]. As the origin of goat domestication, the Middle East has a relatively high nucleotide diversity in its population, which was also verified in this study [20]. In LD analysis, Huai goat and Boer goat were distinguished from other breeds. It’s probably more manual selection. Henan province as an important commercial and transportation area may promote the occurrence of strong selection. As a world famous mutton breed, Boer goat has been introduced by various countries to improve the local breed, which may also be the reason for its rapid LD attenuation.

The characterization of population structure and genetic diversity can help us to evaluate goat genetic resources and play an important role in future utilization and conservation. In this study, the population genetic structure of the BoHuai goat was studied in the background with different goat breeds. As can be seen from the ADMIXTURE analysis (Fig. 1),the ancestry of the Boer goat is mainly from the Boer goat (~ 90%). Therefore, the genetic relationship between BoHuai goats and Boer goats is closer than that of Huai goats. Interestingly, the Huai goat has a mixed pedigree, which is quite different from goat breeds in other parts of China. This suggests that the formation history of the Huai goat may be complicated. We speculate that the unique location of Henan Province may result in more artificial selection of Huai goat, which leads to its complex genetic background, which is similar to that of Nanyang cattle [21]. Notably, In the ADMIXTURE analysis, when K = 2, showde that Boer and African goats share the same genetic background. When K = 3, Boer and African goats showed significant genetic heterogeneity (Fig. S4). This indicates that Boer goats have experienced more artificial selection during breeding.

In our analysis, Boer goat and Huai goat, as the male and female parents of Bohuai goat, have a very close relationship with each other. In order to understand their genetic differences, In order to understand the genetic differences between the two breeds, we performed the GO enrichment analysis of genes harboring > 5 specific nsSNPs. Most of the genes are concentrated in amino acid transport and “calcium ion binding”,It reflects the strong production performance of Bohuai goat. In addition, we also identified significant signatures of selective sweeps in Bohuai goat. After more than 20 years of breeding, the production performance of Bohuai goat has been significantly improved. Fat content is an important factor determining mutton quality. BoHuai goat genome showed signs of selection in some genes of the “Cholesterol metabolism” pathway (LDLR, STAR, ANGPTL8), which plays an important role in lipid metabolism. This may be an important factor leading to fat deposition in Bohuai goats. In order to better understand the relationship between these genes and the excellent traits of Bohuai goats,we looked at the biological function of these genes. For example, the *LDLR* gene regulating cholesterol homeostasis is related to atherosclerosis [22, 23]. The *STAR* (Steroidogenic acute regulatory protein) gene plays an important role in regulating the rate-limiting step in steroid hormone synthesis, and cholesterol side-chain cleavage [24]; In addition, it has also been reported that it may be associated with high fertility in goats [25]. The *ANGPTL8* (Angiopoietin-like protein 8) gene is an important regulator of metabolic disorders [26], blocking *ANGPTL8* in mice promoted triglyceride clearance, energy expenditure, and weight loss [27]. In addition, *ANGPTL8* regulates adipocyte differentiation and adipogenesis in bovines [28]. Therefore, these three genes are likely to play an important role in the growth and development of Bohuai goat population. Meanwhile,We observed a significant peak in the BTA7:9.4–9.9 Mb region. This region contains multiple genes. This region contains 79 candidate genes that were selected by both θπ and CLR methods (Fig. 3B). Therefore, the research on genetic improvement of Bohuai goat genome can focus on this region.

Strong disease resistance is often an important characteristic of local breeds [29]. After years of breeding, Compared with both Bohuai and Boer goats, the Bohuai goats showed stronger disease resistance Many of the candidate genes that we found in the Bohuai goat are related to immunity. For example, functional enrichment analysis showed significant immune-related GO term “positive regulation of T cell proliferation”, including *CD274*, *DHPS*, and *PDCD1LG2* genes. Related studies have shown that the *CD274* gene inhibits host immunity in T lymphocytic proliferative diseases [30]. In cattle, *CD274* was found to be the target of host-targeted therapy in cattle infected with *Mycoplasma Bovis* [31]. It’s likely that the gene also plays an important role in disease resistance in goats. The DHPS gene is associated with antimalarial resistance [32]. Previous pain-related studies in goats have shown that CCL27 is a candidate gene for an immune response [33]. These genes may be related to the higher reproductive rate of Bohuai goats.

## Conclusions

Our genomic analysis provided new insights into the diversity and selection signals of BoHuai goats and their relationship with other breeds of goats. The discovery of genomic diversity will provide a basis for the conservation and the utilization of genetic resources of BoHuai goats. In addition, we identified a series of genes that may play an important role in lipid metabolism and immune response of this breed. These results will provide information for further study on the formation mechanism of various fine traits of Bohuai goats, and also provide reference for molecular breeding of other breeds.

## Methods

### Samples and whole-genome sequencing

In this study, 30 Bohuai goats and 5 Huai goats from Shenqiu County Bohuai Goat breeding farm as samples (female). The goats were immobilized and jugular blood samples were collected using EDTA-K2 anticoagulant tubes and stored in cold storage. Genomic DNA was extracted using the standard phenol-chloroform method [34]. We used Nanodrop to measure the purity of DNA and established libraries of DNA samples with a concentration of more than 1.5 μg. A paired-end library with an average insert length of 500 bp and an average read length of 150 bp was constructed for each individual. Sequencing was performed using Illumina 2000 instruments at the Novogene Bioinformatics, Beijing, China.

To better analyze the genetic diversity and selection signals of Bohuai goats, we also used data from 126 published goats from around the world. These include goats from Europe (*n* = 15, including the Alpine Goat, Italy Goat, and Saanen Goat), African goats (*n* = 16, including the Morocco Black Goat, the Morocco Draa Goat, Morocco indigenous population), goats from the Middle East (*n* = 15, Iran indigenous population), goats from different regions of China (*n* = 73, including Anhui White Goat, Bange Cashmere Goat, Chengde Hornless Goat, Chengdu Grey Goat, Chinese Nubian Goat, Er Lang Shan Cashmere Goat, Erdos Cashmere Goat, Guishan Black Goat, Guizhou Black Goat, JianChangBlack Goat, Jining Grey Goat, Laiwu Black Goat, Leizhou Black Goat, Liaoning Cashmere Goat, Longlin Goat, LVLIANG Black Goat, Maguan Hornless Goat, Matou Goat, Nubian-Longlin Goat, Qinghai Tibetan Goat, Raoshan White Goat, Ritu Cashmere Goat, Shannan White Goat, The Tibetan Goat, Wu Zhu Mu Qin White Goat, Xiangdong Black Goat, Xinjiang Goat, Yimeng Black Goat, Yudong White Goat, Yunnanblack Goat, Zhongwei Goat) and seven Boer goats. Therefore, a total of 161 goats were used in this study.

### Alignment and SNP calling

Trimmomatic software (v0.36) was used to trim raw sequence reads with the parameters: “LEADING:20, TRAILING:20, SLIDINGWINDWOE: 3:15, AVGQUAL:20, MINLEN:35, TOPHRED33”. The cleans reads were aligned to the goat reference assembly ARS1 by Sentieon software [18]. Then Single nucleotide polymorphisms (SNPs) were detected by the Genome Analysis Toolkit (GATK, version 4.1.8.1) with diverse modules [35]. The raw SNPs were called using the “SelectVariants” module of GATK. After SNPs were called, we used the “VariantFiltration” module to filter the raw SNPs with the following parameters: QD < 2.0, QUAL < 30.0, SOR > 3.0, FS > 60.0, MQ < 40.0, MQRankSum < − 12.5, and ReadPosRankSum < − 8.0. VCFtools were used to remove the variants with a minor allele frequency (MAF) lower than 0.05 and variants with more than 0.1 missing genotypes at the meta-population level. Finally, biallelic SNPs were extracted and used in the subsequent analyses. Annovar software was used to annotate the functions of each SNP.

### Population genetic analysis

The SNPs in high levels of pair-wise LD were pruned by Plink [36] with the parameter (−-inder-pair-wise 50 5 0.2) to perform ADMIXTURE analysis. Principal component analysis (PCA) was performed using the SmartPCA program in the Eigensoft V5.0 package [37]. ADMIXTURE v1.3 [38] was used for population structure analysis, the kinship set is from 2 to 12 (Table S4). We used PLINK software to make the matrix of pairwise genetic distances and then it was used for constructing an unrooted evolutionary tree. The visualization of the evolutionary tree was done by MEGA7 [39] and embellished by FigTree (http://tree.bio.ed.ac.uk/software/figtree/).

### Selective sweep identification

In order to identify the selection signatures that are driven by artificial selection and genetic adaptation to the local environment, more than two strategies were used to scan the genomes of Bohuai goats. The nucleotide diversity and the composite likelihood ration (CLR) [40] method were used in the Bohuai goat population. First, the SNP loci with allele frequency less than 0.05 were removed. After that, we used VCFtools [41] to estimate the nucleotide diversity by a sliding window approach in which widows are 50kbs and the step is 20 kb. The SweepFinder2 [41] was used for calculating the CLR for sites in non-overlapping 50 kb windows. We calculate the empirical *P* values of π windows and CLR windows, and select the overlapping region of the top 1% windows of each method as a candidate signal. In addition, fixation index (Fst) and cross-population extended haplotype homozygosity (XP-EHH) were used for comparing Boer goat and Huai goat. We used VCFtools to analyze *F*_ST_ with a 50 kb window and a 20 kb step. SELSCAN V1.1 [42] was used to calculate the XP-EHH statistics for each population based on extended haplotypes. For XP-EHH selection scans, our test statistics are the average of standardized XP-EHH scores for each 50 KB region. The XP-EHH score is directional: a positive score indicates that selection may have occurred in Boer goats, whereas a negative score indicates that selection may have occurred in the reference population. *P*-value < 0.01 was used as a threshold for significant genomic regions. Genomic regions identified by two methods are considered candidates for positive selection. To obtain a better understanding of the function and signaling pathways of the candidate genes, the GO and KEGG pathways were enriched using KOBAS 3.0 [43].

## Supplementary Information

**Additional file 1: Figure S1.** SNP intersection of BoHuai goat with Boer goat (male parent) and Huai goat (female parent).

**Additional file 2: Figure S2.** Specific nsSNP gene enrichment analysis in Boer goat.

**Additional file 3: Figure S3.** Specific nsSNP gene enrichment analysis in Huai goat.

**Additional file 4: Figure S4.** ADMIXTURE was used with K = 2–8 for model-based clustering among different goat.

**Additional file 5: Table S1.** Summary of sequencing data. **Table S2.** List of additional goat samples for analysis of genetic background. **Table S3.** Distribution of SNPs identified in goat breeds within various genomic regions annotated by ANNOVAR. Only the breeds with a sample size no less than 5 were calculated in the table. **Table S4.** CV error corresponding to different K values. **Table S5.** GO enrichment results for the genes containing specific nsSNPs > 5 in Boer goat. **Table S6.** GO enrichment results for the genes containing specific nsSNPs > 5 in Huai goat. **Table S7.** A summary of genes from θπ in BoHuai goat. **Table S8.** A summary of genes from CLR in BoHuai goat. **Table S9.** KEGG pathway analysis of BoHuai goat candidate genes overlapped by θπ and CLR methods. **Table S10.** GO enrichment of BoHuai goat candidate genes overlapped by θπ and CLR methods. **Table S11.** A summary of genes from Fst between Boer and Huai goat. **Table S12.** Summary of genes screened by XP-EHH method between Boer and Huai goat. **Table S13.** KEGG pathway analysis of candidate genes overlapped by Fst and XP-EHH methods. **Table S14.** Go enrich analysis of candidate genes overlapped by Fst and XP-EHH methods.

## Data Availability

The datasets generated and analysed during the current study are available in the GenBank epository. Bioproject accession number is PRJNA699447.

## References

[1] Zeder MA, Hesse B (2000). The initial domestication of goats (Capra hircus) in the Zagros mountains 10,000 years ago. Science.

[2] Luikart G (2001). Multiple maternal origins and weak phylogeographic structure in domestic goats. Proc Natl Acad Sci.

[3] Manceau V (1999). Systematics of the genus Capra inferred from mitochondrial DNA sequence data. Mol Phylogenet Evol.

[4] Takada T (1997). Bezoar (Capra aegagrus) is a matriarchal candidate for ancestor of domestic goat (Capra hircus): evidence from the mitochondrial DNA diversity. Biochem Genet.

[5] Zeder MA (2008). Domestication and early agriculture in the Mediterranean Basin: origins, diffusion, and impact. Proc Natl Acad Sci U S A.

[6] Zheng Z (2020). The origin of domestication genes in goats. Sci Adv.

[7] Lai F-N (2016). Whole-genome scanning for the litter size trait associated genes and SNPs under selection in dairy goat (Capra hircus). Sci Rep.

[8] Li R (2020). Genome-wide scan of selection signatures in Dehong humped cattle for heat tolerance and disease resistance. Anim Genet.

[9] Wang X (2016). Whole-genome sequencing of eight goat populations for the detection of selection signatures underlying production and adaptive traits. Sci Rep.

[10] Xu Z (2020). Copy number variation of CADM2 gene revealed its association with growth traits across Chinese Capra hircus (goat) populations. Gene.

[11] Rupp R (2016). Genomic application in sheep and goat breeding. Animal Front.

[12] Ng PC, Kirkness EF. Kirkness, Whole genome sequencing. Genetic variation: Methods and protocols. 2010. p. 215–26. 10.1007/978-1-60327-367-1_12 20238084

[13] Friedenberg SG, Meurs KM, Mackay TFC (2016). Evaluation of artificial selection in standard poodles using whole-genome sequencing. Mamm Genome.

[14] Skinner JD (1972). Utilisation of the Boer goat for intensive animal production. Trop Anim Health Prod.

[15] Barry D, Godke R (1991). The Boer goat: the potential for cross breeding. Proceedings of the National symposium on goat meat production and marketing.

[16] Wenying H (2008). Study on the meat quality and antioxidant function of crossbreed F~ 1 between Boer goat and Huai goat. J Anhui Agric Sci.

[17] Guizhi Z (2007). Study on mutton performance and quality of hybrids between Boer and Huai goat. J Anhui Agric Sci.

[18] Weber JA (2016). Sentieon DNA pipeline for variant detection-software-only solution, over 20× faster than GATK 3.3 with identical results. PeerJ PrePrints.

[19] Brito LF (2017). Genetic diversity and signatures of selection in various goat breeds revealed by genome-wide SNP markers. BMC Genomics.

[20] Daly KG (2018). Ancient goat genomes reveal mosaic domestication in the Fertile Crescent. Science.

[21] Mei C (2018). Genetic architecture and selection of Chinese cattle revealed by whole genome resequencing. Mol Biol Evol.

[22] Go G-w, Mani A (2012). Low-density lipoprotein receptor (LDLR) family orchestrates cholesterol homeostasis. Yale J Biol Med.

[23] Véniant MM, Withycombe S, Young SG (2001). Lipoprotein size and atherosclerosis susceptibility in Apoe−/− and Ldlr−/− mice. Arterioscler Thromb Vasc Biol.

[24] Sugawara T (1997). Regulation of expression of the steroidogenic acute regulatory protein (StAR) gene: a central role for steroidogenic factor 1. Steroids.

[25] Zi XD (2018). Comparative analysis of ovarian transcriptomes between prolific and non-prolific goat breeds via high-throughput sequencing. Reprod Domest Anim.

[26] Luo M, Peng D (2018). ANGPTL8: an important regulator in metabolic disorders. Front Endocrinol.

[27] Gusarova V (2017). ANGPTL8 blockade with a monoclonal antibody promotes triglyceride clearance, energy expenditure, and weight loss in mice. Endocrinology.

[28] Wei X (2019). ANGPTL8 regulates adipocytes differentiation and adipogenesis in bovine. Gene.

[29] Ma Q (2014). Tissue specificity and species superiority of cathelicidin gene expression in Chinese indigenous min pigs. Livest Sci.

[30] Wilcox RA (2009). B7-H1 (PD-L1, CD274) suppresses host immunity in T-cell lymphoproliferative disorders. Blood.

[31] Rambault M (2021). Neutrophils encompass a regulatory subset suppressing T cells in apparently healthy cattle and mice. Front Immunol.

[32] Gesase S (2009). High resistance of plasmodium falciparum to sulphadoxine/pyrimethamine in northern Tanzania and the emergence of dhps resistance mutation at codon 581. PLoS One.

[33] Deng X, et al. Identification of key genes and pathways involved in response to pain in goat and sheep by transcriptome sequencing. Biol Res. 2018;51. 10.1186/s40659-018-0174-7 PMC6098572 30119702

[34] Hogan B, Costantini F, Lacy E (1986). Manipulating the mouse embryo: a laboratory manual.

[35] Van der Auwera GA (2013). From FastQ data to high-confidence variant calls: the genome analysis toolkit best practices pipeline. Curr Protoc Bioinformatics.

[36] Purcell S (2007). PLINK: a tool set for whole-genome association and population-based linkage analyses. Am J Hum Genet.

[37] Patterson N, Price AL, Reich D (2006). Population structure and eigenanalysis. PLoS Genet.

[38] Alexander DH, Lange K (2011). Enhancements to the ADMIXTURE algorithm for individual ancestry estimation. BMC Bioinformatics.

[39] Kumar S, Stecher G, Tamura K (2016). MEGA7: molecular evolutionary genetics analysis version 7.0 for bigger datasets. Mol Biol Evol.

[40] Nielsen R (2005). Genomic scans for selective sweeps using SNP data. Genome Res.

[41] Danecek P (2011). The variant call format and VCFtools. Bioinformatics.

[42] Szpiech ZA, Hernandez RD (2014). Selscan: an efficient multithreaded program to perform EHH-based scans for positive selection. Mol Biol Evol.

[43] Xie C (2011). KOBAS 2.0: a web server for annotation and identification of enriched pathways and diseases. Nucleic Acids Res.

