# Supplementary figures and images for: Genetic diversity and signatures of selection in BoHuai goat revealed by whole-genome sequencing
Source: BMC Genomics. 2023 Mar 15;24:116. doi: 10.1186/s12864-023-09204-9 (PMC10018941; doi:10.1186/s12864-023-09204-9)

Boer goat

BoHuai goat

2301590

2925755

1077304

9903772

1096438

940920

2680781

Huai goat

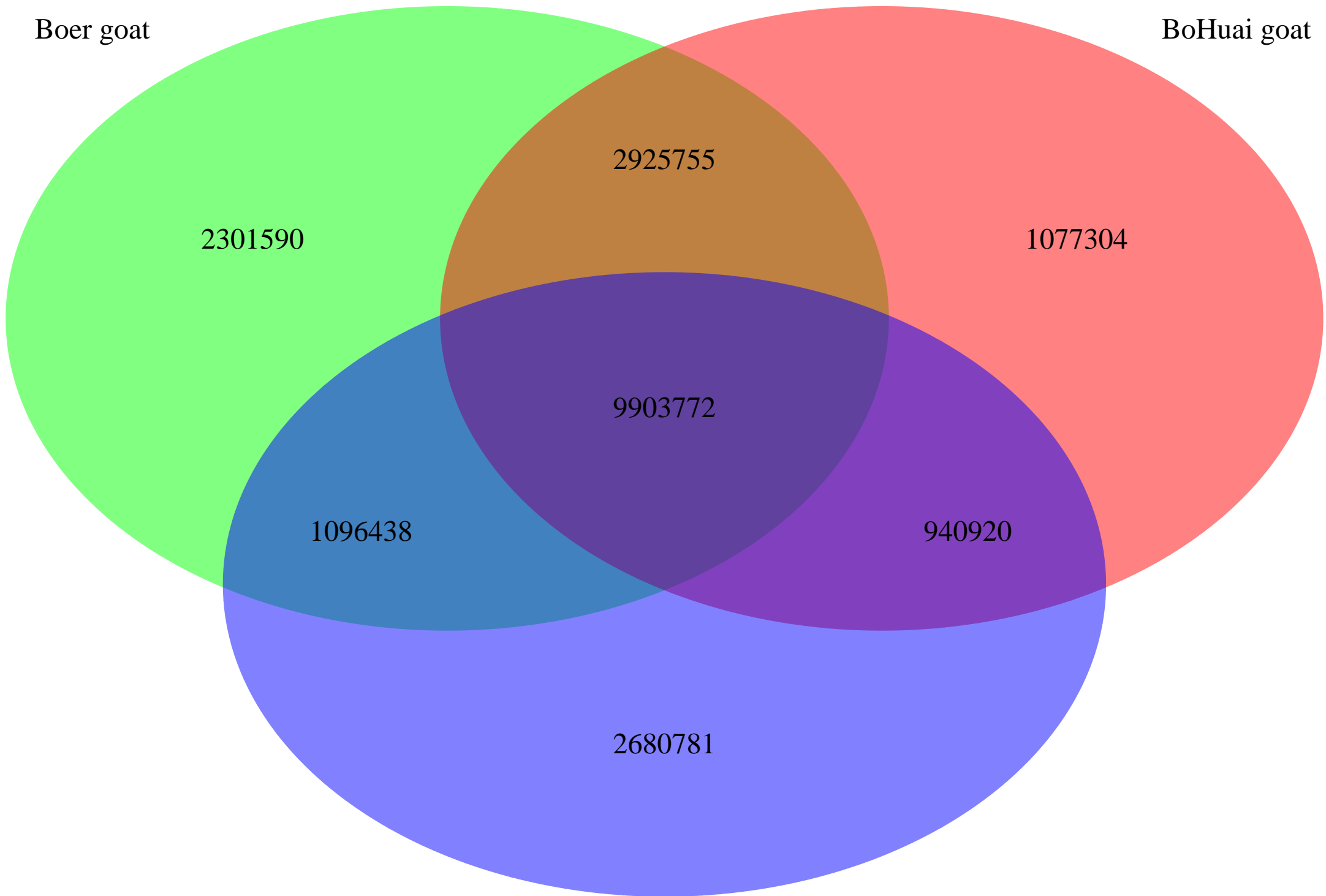

Supplement: Supplementary file 2 — Additional file 2: Figure S2. Specific nsSNP gene enrichment analysis in Boer goat. [file 12864_2023_9204_MOESM2_ESM.pdf]

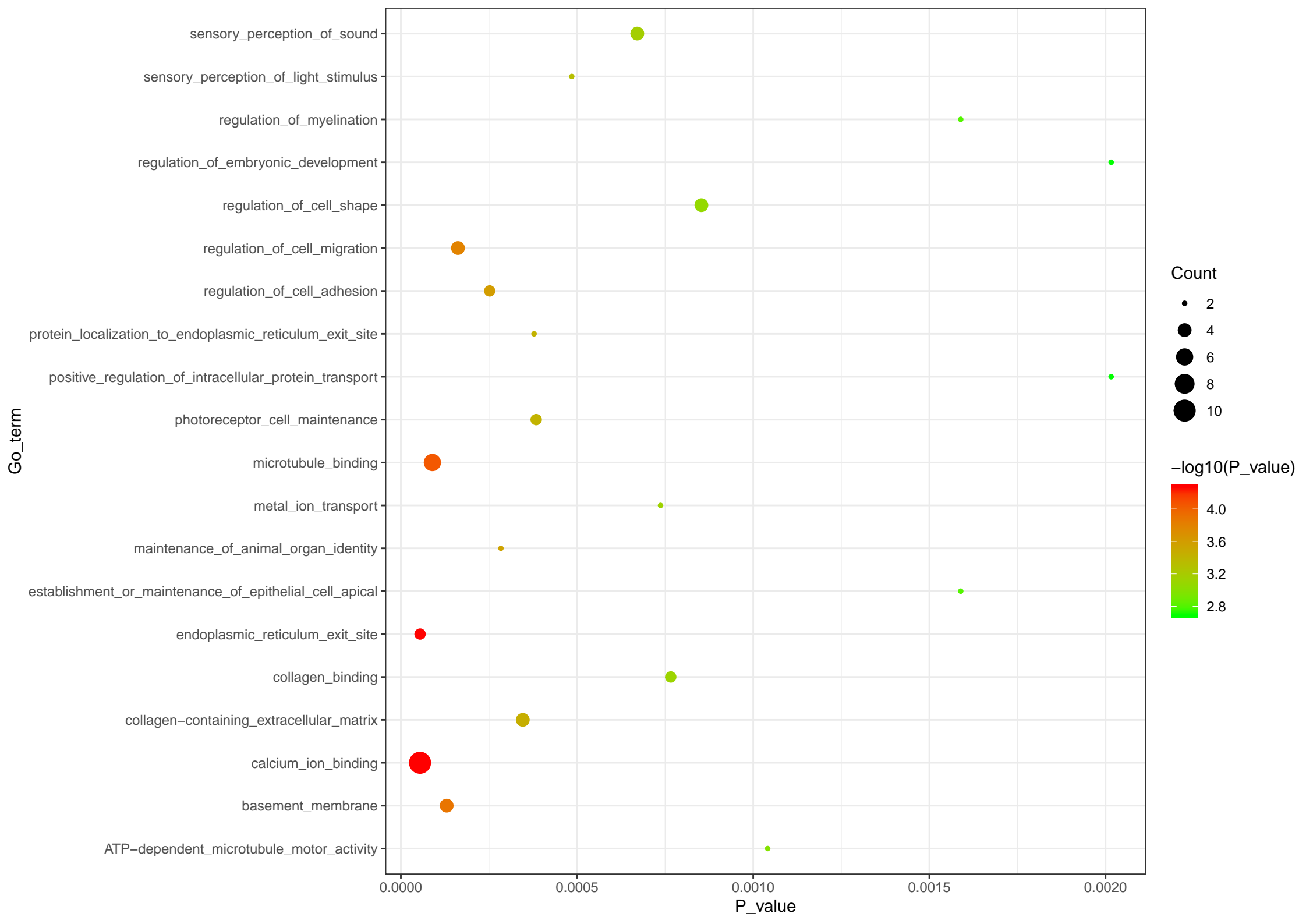

Supplement: Supplementary file 3 — Additional file 3: Figure S3. Specific nsSNP gene enrichment analysis in Huai goat. [file 12864_2023_9204_MOESM3_ESM.pdf]

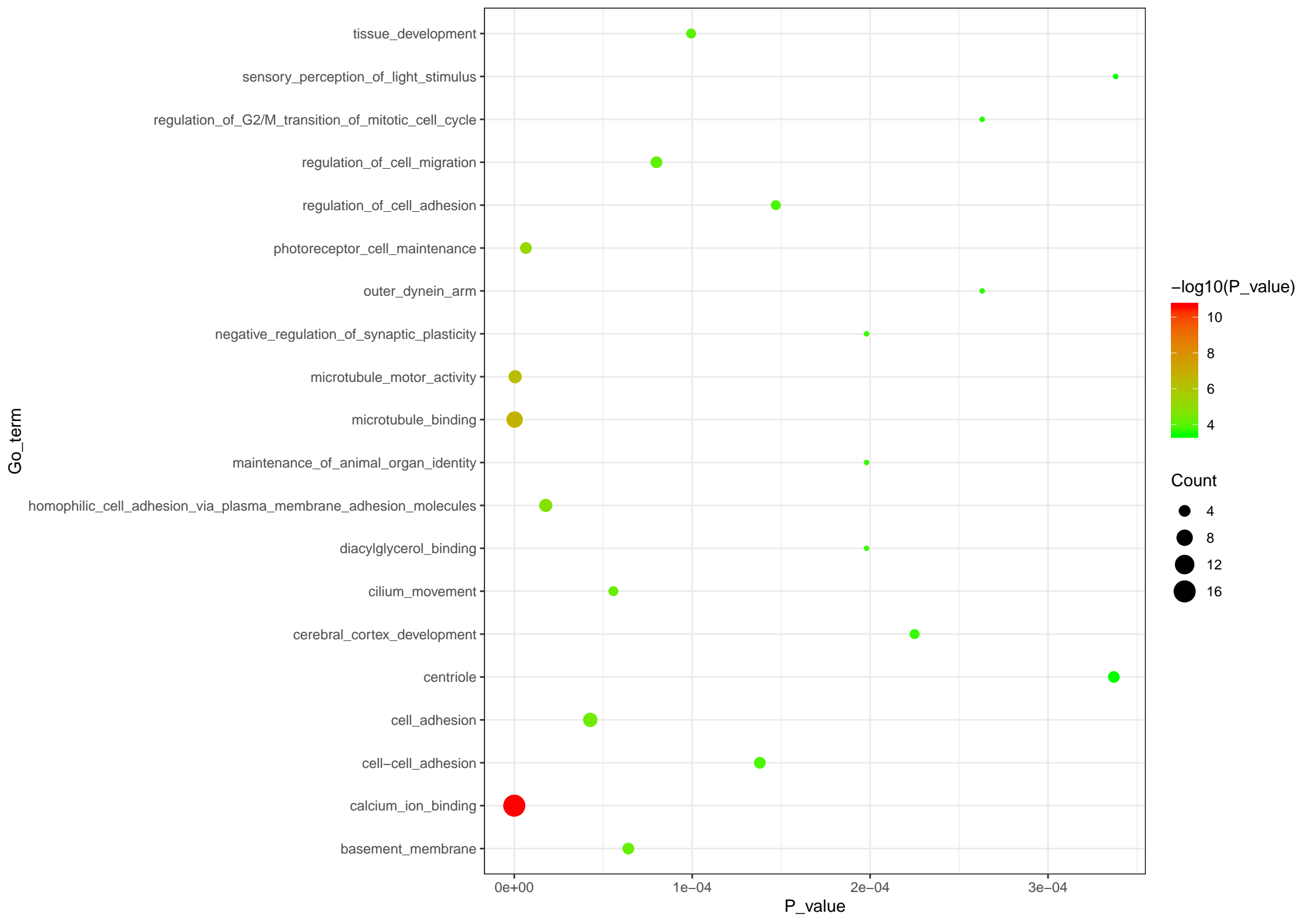

Supplement: Supplementary file 4 — Additional file 4: Figure S4. ADMIXTURE was used with K = 2–8 for model-based clustering among different goat. [file 12864_2023_9204_MOESM4_ESM.pdf]

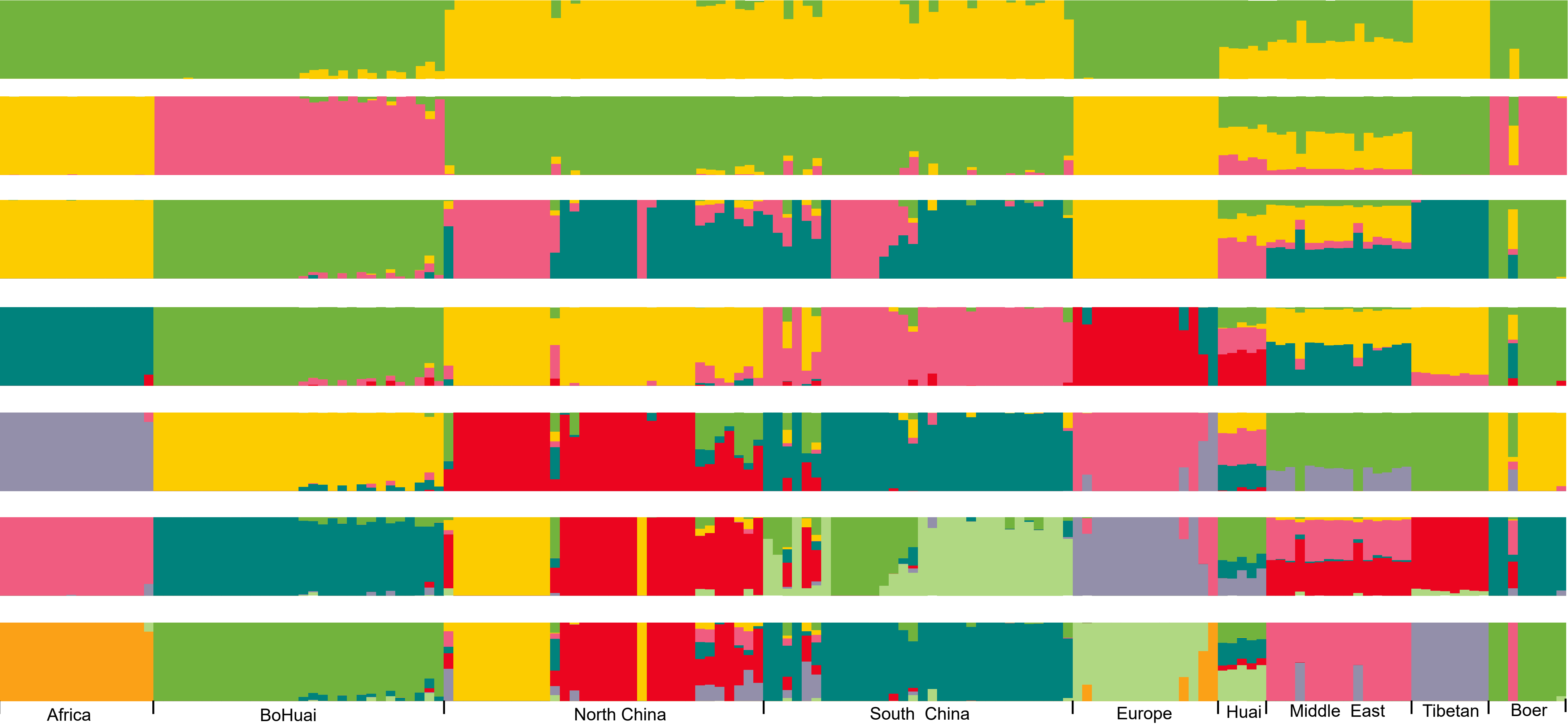

Supplement: Supplementary file 5 — Additional file 5: Table S1. Summary of sequencing data. Table S2. List of additional goat samples for analysis of genetic background. Table S3. Distribution of SNPs identified in goat breeds within various genomic regions annotated by ANNOVAR. Only the breeds with a sample size no less than 5 were calculated in the table. Table S4. CV error corresponding to different K values. Table S5. GO enrichment results for the genes containing specific nsSNPs > 5 in Boer goat. Table S6. GO enrichment results for the genes containing specific nsSNPs > 5 in Huai goat. Table S7. A summary of genes from θπ in BoHuai goat. Table S8. A summary of genes from CLR in BoHuai goat. Table S9. KEGG pathway analysis of BoHuai goat candidate genes overlapped by θπ and CLR methods. Table S10. GO enrichment of BoHuai goat candidate genes overlapped by θπ and CLR methods. Table S11. A summary of genes from Fst between Boer and Huai goat. Table S12. Summary of genes screened by XP-EHH method between Boer and Huai goat. Table S13. KEGG pathway analysis of candidate genes overlapped by Fst and XP-EHH methods. Table S14. Go enrich analysis of candidate genes overlapped by Fst and XP-EHH methods. [file 12864_2023_9204_MOESM5_ESM.pdf]
